# Maternal RSVpreF and Infant Nirsevimab Immunizations Uptake During Respiratory Syncytial Virus Season

**DOI:** 10.1001/jamanetworkopen.2024.60729

**Published:** 2025-02-19

**Authors:** Ethan A. Litman, Tina Yi Jin Hsieh, Anna M. Modest, Kathleen Clarke, Melissa Dzinoreva, Valerie Perrinez, Esther Apraku Bondzie, Lydia Gallup, Eleanor Schonberg, Marjorie Rowe, K’ara Locke, Oluwaseyi Oginni, Robert Jones, Chloe A. Zera, Ai-ris Y. Collier

**Affiliations:** 1 Department of Obstetrics and Gynecology, Beth Israel Deaconess Medical Center, Boston, Massachusetts; 2 Department of Obstetrics, Gynecology, and Reproductive Biology, Harvard Medical School, Boston, Massachusetts; 3 Center for Virology and Vaccine Research, Beth Israel Deaconess Medical Center, Boston, Massachusetts

## Abstract

This cohort study evaluates the use rate of respiratory syncytial virus (RSV) vaccines to prevent RSV-related lower respiratory tract disease among infants.

## Introduction

Respiratory syncytial virus (RSV) is a common virus predominately resulting in mild cold or flu-like symptoms for most adults and children.^1^ In older adults and infants, infection is a major cause of hospitalization and morbidity. In 2023, the Centers for Disease Control and Prevention (CDC) Advisory Committee on Immunization Practice recommended the RSVpreF (Pfizer) vaccination during pregnancy to prevent infant RSV–related lower respiratory tract disease or nirsevimab administration to infants younger than 8 months born during the RSV season to a mother who is unvaccinated or vaccinated less than 14 days before delivery.^2,3^ These 2 new RSV interventions and historically disparate vaccine distribution in pregnancy presented an opportunity to study their simultaneous uptake.^4^ We described RSVpreF vaccination and nirsevimab administration patterns during the 2023 to 2024 RSV season.

## Methods

This retrospective cohort study included pregnant individuals and their infants delivered between September 1, 2023, and January 31, 2024, at an urban academic center. The Beth Israel Deaconess Medical Center Institutional Review Board deemed this study exempt from review and waived informed consent because data use was for purposes of health care operations. We followed the STROBE reporting guideline.

Maternal and infant demographics and clinical information were abstracted from the center’s electronic health record. Race and ethnicity were self-reported (eTable in Supplement 1) and collected to ensure generalizability of findings. RSVpreF and nirsevimab immunizations were available at the beginning of RSV season. RSVpreF and nirsevimab administration timing, location, and ordering clinician were abstracted from a statewide immunization portal maintained by the Massachusetts Department of Public Health. Analysis was performed using Python 3.13.1 (Python Software Foundation).

## Results

During the study period, 1899 pregnant patients (median [IQR] age, 33.9 [30.9-36.8] years) were admitted for childbirth. Of 1955 associated fetuses or neonates, 15 were excluded due to abortions and perinatal demise, leaving 1940 live births (median [IQR] gestational age, 39.1 [37.7-39.9] weeks) for inclusion. Most mothers received care in an academic (952 of 1899 [50.1%]) or private practice (721 [38.0%]) and had commercial insurance (1290 [67.9%]). The preterm birth rate was 12.5% (236) at less than 37 weeks and 3.0% (57) at less than 32 weeks (Table).

**Table.** Maternal and Infant Characteristics During the 2023 to 2024 Respiratory Syncytial Virus Season

Characteristics	Sample, No. (%)				
	RSVpreF	Nirsevimab	Dual immunization	Unvaccinated	Overall
Pregnant patients (% of pregnancies)	361 (19.0)	637 (33.5)	28 (1.5)	873 (45.9)	1899 (100)
Maternal age, median (IQR), y	34.8 (32.1-37.5)	33.6 (30.6-36.7)	34.7 (31.6-36.4)	33.7 (30.6-36.6)	33.9 (30.9-36.8)
Race and ethnicity^a^					
Asian, non-Hispanic	60 (16.6)	96 (15.1)	5 (17.9)	91 (10.4)	252 (13.3)
Black, non-Hispanic	20 (5.5)	77 (12.1)	4 (14.3)	120 (13.7)	221 (11.6)
Hispanic or Latinx	22 (6.1)	81 (12.7)	2 (7.1)	95 (10.9)	200 (10.5)
White, non-Hispanic	187 (51.8)	243 (38.1)	8 (28.6)	390 (44.7)	828 (43.6)
Other or multiracial^b^	21 (5.8)	44 (6.9)	4 (14.3)	45 (5.2)	114 (6.0)
Unknown	51 (14.1)	96 (15.1)	5 (17.9)	132 (15.1)	284 (15.0)
Payer type					
Commercial	287 (79.5)	402 (63.1)	21 (75.0)	580 (66.4)	1290 (67.9)
Government	74 (20.5)	235 (36.9)	7 (25.0)	293 (33.6)	609 (32.1)
Obstetric practice setting					
Academic practice	108 (29.9)	349 (54.8)	10 (35.7)	485 (55.6)	952 (50.1)
Community health center	50 (13.9)	80 (12.6)	3 (10.7)	93 (10.7)	226 (11.9)
Private practice	203 (56.2)	208 (32.7)	15 (53.6)	295 (33.8)	721 (38.0)
Plurality					
Singleton	355 (98.3)	611 (95.9)	26 (92.9)	854 (97.8)	1846 (97.2)
Twins	6 (1.7)	23 (3.6)	2 (7.1)	19 (2.2)	50 (2.6)
Triplets	0	3 (0.5)	0	0	3 (0.2)
Parity					
Nulliparous	175 (48.5)	292 (45.8)	16 (57.1)	387 (44.3)	930 (49.0)
Multiparous	186 (51.5)	345 (54.2)	12 (42.9)	486 (55.7)	969 (51.0)
Non–live births					
Spontaneous abortion	0	0	0	4 (0.5)	4 (0.2)
Stillbirths ≥20 wk	0	0	0	3 (0.3)	3 (0.2)
Neonatal demise^c^	0	0	0	1 (0.1)	1 (0.1)
Induction termination	0	0	0	7 (0.8)	7 (0.3)
Preterm births^d^					
<37 wk	22 (6.0)	108 (17.0)	10 (35.7)	96 (11.1)	236 (12.5)
<32 wk	0	28 (4.4)	0	28 (3.4)	57 (3.0)
Time from maternal vaccination to birth, median (IQR), d	32.0 (23.0-40.0)	NA	10.0 (3.0-14.2)	NA	31.0 (21.0-39.9)
No. of live-born infants (%)	367 (18.9)	666 (34.3)	30 (1.5)	877 (45.2)	1940 (100)
Gestational age at birth, median (IQR), wk	39.1 (38.3-39.7)	39.0 (37.3-39.9)	37.3 (35.8-37.9)	39.1 (37.9-40.0)	39.1 (37.7-39.9)
Birthweight, median (IQR), g	3300 (2970-3615)	3153 (2760-3510)	2743 (2569-3213)	3280 (2960-3615)	3240 (2900-3584)

Abbreviation: NA, not applicable.

^a^ eTable in Supplement 1 provides complete information on self-reported race and ethnicity.

^b^ Self-reported Middle Eastern, Native Hawaiian or Other Pacific Islander, or other race and ethnicity were categorized as other or multiple.

^c^ Neonatal demise due to previable birth at 20 weeks 6 days.

^d^ Excluding terminations and spontaneous abortions before 20 weeks.

Most live-born infants received RSV protection (1063 of 1940 [54.8%]), of whom 696 (65.5%) received protection after birth. Thirty infants received both maternal and newborn protection, of whom 19 (63.3%) appropriately received nirsevimab, with birth occurring less than 14 days after maternal vaccination. The preterm birth rate observed for mothers who received RSVpreF was 8.2% (32 of 389).

At RSV season end, 389 patients (20.5%) received RSVpreF and 665 infants (35.0%) received nirsevimab. The proportion of infants receiving RSV protection increased each month (eg, 27.1% in September 2023; 77.7% in January 2024) (Figure, A). Differences in maternal vaccine rates across race and ethnicity categories persisted throughout the RSV season; however, race-based disparities in nirsevimab administration decreased (Table and Figure, B). RSVpreF was administered mostly at outpatient commercial pharmacies to mothers with commercial insurance (202 of 308 [65.6%]) or government insurance (42 of 81 [51.9%]). RSVpreF vaccination rates improved over time through increasing administration at both prenatal offices and outpatient pharmacies (Figure, C). Most newborns were immunized during the birth hospitalization (Figure, D).

**Figure.**
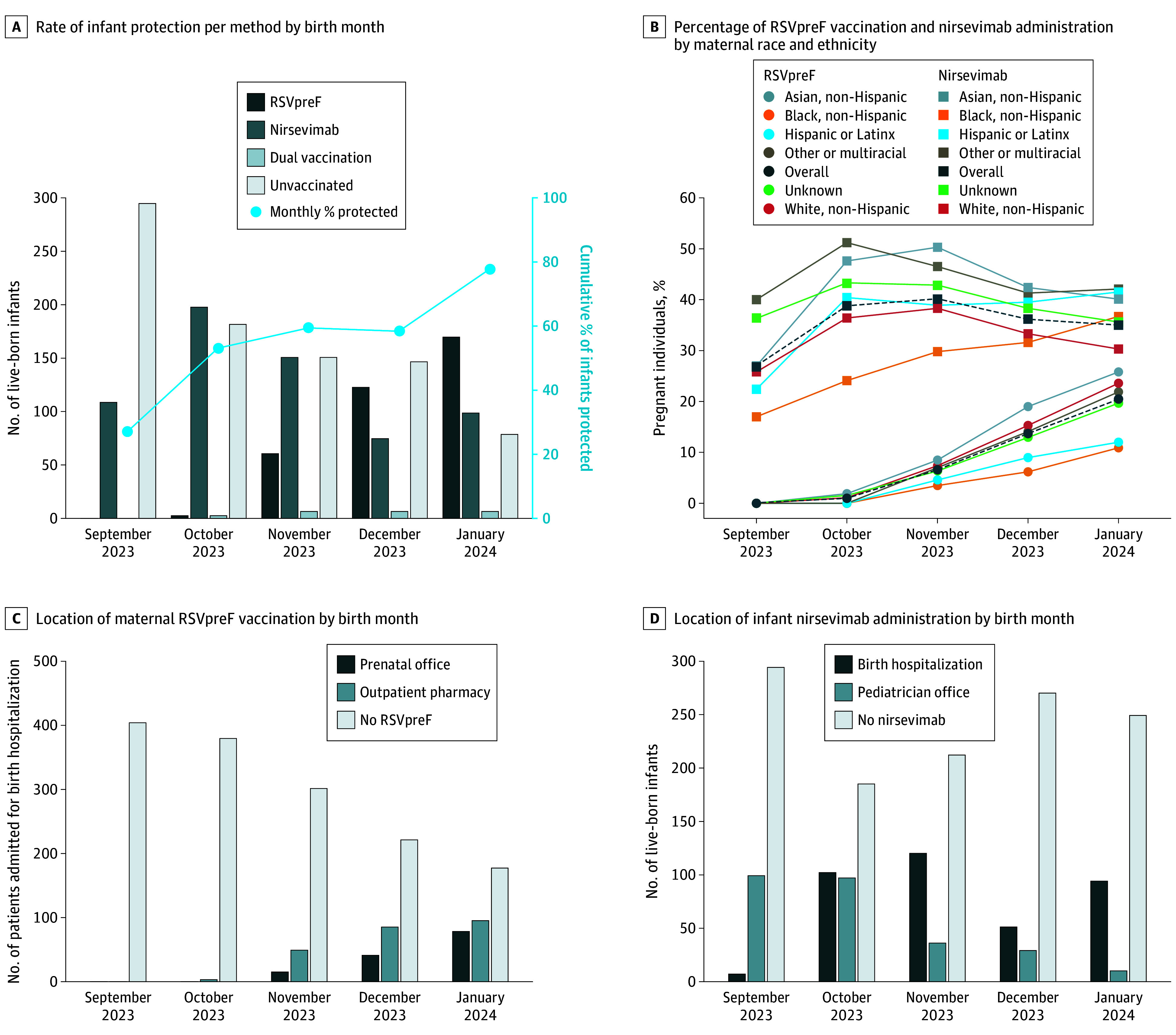
Infant Immunization Status and Location by Birth Month Ascertained at the End of the 2023 to 2024 Respiratory Syncytial Virus (RSV) Season The number of pregnant individuals who delivered between September 1, 2023, and January 31, 2024, was 1899. The number of live-born infants born between September 1, 2023, and January 31, 2024, was 1940.

## Discussion

Overall, 20.5% of mothers and 35.0% of infants received RSV immunizations, consistent with previous CDC report^5^ but lower than the 35% maternal vaccination rate reported in a New York health system.^6^ Increases in both pharmacy- and office-administered immunizations during hospitalization were associated with enhanced RSV protection. Disparities in maternal, but not infant, vaccination persisted throughout the study.

Study strengths were the large, diverse patient population with varied practice settings and payer types. Immunization data were uniformly collected using a statewide mandatory vaccine reporting data source. Study limitations were lack of data on patient decision-making regarding vaccination and potential for vaccination-status misclassification. Future research should focus on systemic reasons for nonvaccination. Health care systems should focus on infant immunization during the birth hospitalization to increase equity.

## Notes

Supplement 1. **eTable.** Statement on Self-Reported Race and Ethnicity

Supplement 2.
Data Sharing Statement
