# Supplementary material for: Maternal RSVpreF and Infant Nirsevimab Immunizations Uptake During Respiratory Syncytial Virus Season
Source: JAMA Netw Open. 2025 Feb 19;8(2):e2460729. doi: 10.1001/jamanetworkopen.2024.60729 (PMC11840643; doi:10.1001/jamanetworkopen.2024.60729)
Supplement: Supplement 1. — eTable. Statement on Self-Reported Race and Ethnicity [file jamanetwopen-e2460729-s001.pdf]

Supplemental Online Content

Litman EA, Hsieh TYJ, Modest AM, et al. Maternal RSVpreF and infant nirsevimab immunizations uptake during respiratory syncytial virus season. *JAMA Netw Open*. 2025;8(2):e2460729. doi:10.1001/jamanetworkopen.2024.60729

eTable. Statement on Self-Reported Race and Ethnicity

This supplemental material has been provided by the authors to give readers additional information about their work.

© 2025 Litman EA et al. *JAMA Network Open*

eTable. Statement on Self-Reported Race and Ethnicity

Our health system is a large community-based enterprise and subsequently provides care to racially and ethnically diverse patient populations. It is well-documented that healthcare systems often do not have adequate race/ethnicity selections for marginalized and under-represented populations. To reduce under-representation, the electronic health record has over thirty different categories of race and ethnicity. For research purposes, we grouped these sub-categories into larger general categories similar to those reported nationally by the Centers for Disease Control and Prevention in order to highlight disparate trends among patients. Below is a table outlining our methodology for the grouping of racial/ethnic subcategories.

**Sub-Category**	**Broad Category**
ASIAN	Asian, non-Hispanic
ASIAN - ASIAN INDIAN	Asian, non-Hispanic
ASIAN - CAMBODIAN	Asian, non-Hispanic
ASIAN - CHINESE	Asian, non-Hispanic
ASIAN - FILIPINO	Asian, non-Hispanic
ASIAN - JAPANESE	Asian, non-Hispanic
ASIAN - KOREAN	Asian, non-Hispanic
ASIAN - OTHER	Asian, non-Hispanic
ASIAN - VIETNAMESE	Asian, non-Hispanic
BLACK/AFRICAN	Black, non-Hispanic
BLACK/AFRICAN AMERICAN	Black, non-Hispanic
BLACK/CAPE VERDEAN	Black, non-Hispanic
BLACK/HAITIAN	Black, non-Hispanic
HISPANIC OR LATINO	Hispanic / Latinx
HISPANIC/LATINO - CENTRAL AMERICAN (OTHER)	Hispanic / Latinx
HISPANIC/LATINO - COLOMBIAN	Hispanic / Latinx
HISPANIC/LATINO - CUBAN	Hispanic / Latinx
HISPANIC/LATINO - DOMINICAN	Hispanic / Latinx
HISPANIC/LATINO - GUATEMALAN	Hispanic / Latinx
HISPANIC/LATINO - HONDURAN	Hispanic / Latinx
HISPANIC/LATINO - MEXICAN	Hispanic / Latinx
HISPANIC/LATINO - PUERTO RICAN	Hispanic / Latinx
HISPANIC/LATINO - SALVADORAN	Hispanic / Latinx
MIDDLE EASTERN	Other / Multiple
NATIVE HAWAIIAN OR OTHER PACIFIC ISLANDER	Other / Multiple

© 2025 Litman EA et al. *JAMA Network Open*

OTHER	Other / Multiple
PATIENT DECLINED TO ANSWER	Unknown
PORTUGUESE	Hispanic / Latinx
SOUTH AMERICAN	Hispanic / Latinx
UNABLE TO OBTAIN	Unknown
WHITE	White, non-Hispanic
WHITE - BRAZILIAN	Hispanic / Latinx
WHITE - EASTERN EUROPEAN	White, non-Hispanic
WHITE - OTHER EUROPEAN	White, non-Hispanic
WHITE – RUSSIAN	White, non-Hispanic

© 2025 Litman EA et al. *JAMA Network Open*
