# Supplementary material for: Maternal RSVpreF and Infant Nirsevimab Immunizations Uptake During Respiratory Syncytial Virus Season
Source: JAMA Netw Open. 2025 Feb 19;8(2):e2460729. doi: 10.1001/jamanetworkopen.2024.60729 (PMC11840643; doi:10.1001/jamanetworkopen.2024.60729)
Supplement: Supplement 2. — Data Sharing Statement [file jamanetwopen-e2460729-s002.pdf]

Data Sharing Statement

Litman. Maternal RSVpreF and Infant Nirsevimab Immunizations Uptake During Respiratory Syncytial Virus Season. *JAMA Netw Open*. Published February 19, 2025. doi:10.1001/jamanetworkopen.2024.60729

Data
Data available: Yes
Data types: Deidentified participant data
How to access data: Request for data should be sent to acollier@bidmc.harvard.edu
When available: With publication

Supporting Documents
Document types: None

Additional Information
Who can access the data: researchers whose proposed use of the data has been approved
Types of analyses: for research purposes
Mechanisms of data availability: with investigator support and a signed data access agreement
